# Retroperitoneal Extrarenal Angiomyolipomas: An Evidence-Based Approach to a Rare Clinical Entity

**DOI:** 10.1155/2012/374107

**Published:** 2012-07-12

**Authors:** Emmanuel J. Minja, Megan Pellerin, Nicole Saviano, Ronald S. Chamberlain

**Affiliations:** ^1^Department of Surgery, Saint Barnabas Medical Center, Livingston, NJ 07039, USA; ^2^School of Medicine, St. George's University, Grenada; ^3^Department of Pathology, Saint Barnabas Medical Center, Livingston, NJ 07039, USA; ^4^Department of Surgery, University of Medicine and Dentistry of New Jersey, Newark, NJ, USA

## Abstract

Extrarenal angiomyolipomas (ERAMLs) are rare tumors that present as incidentalomas upon imaging for other conditions. Retroperitoneal ERAMLs present a unique challenge from a diagnostic and treatment standpoint as they can mimic other benign and malignant retroperitoneal tumors. We present a case of a 39-year-old female with a 19.3 cm × 13.5 cm × 10.7 cm left extrarenal retroperitoneal mass. Histopathologic examination and HMB-45 staining revealed the mass to be a retroperitoneal ERAML. Our case report provides a comprehensive literature review and an evidence-based algorithm for taking care of patients with ERAMLs.

## 1. Introduction

Extrarenal angiomyolipomas (ERAMLs) represent a very rare subset of tumors that often present as incidentalomas upon imaging for other conditions. Lesions located in the retroperitoneum present a unique challenge from a diagnostic and treatment standpoint as they can mimic other malignant retroperitoneal lesions. We present a case of a 39-year-old female with a 19.3 cm × 13.5 cm × 10.7 cm left extrarenal retroperitoneal mass detected on a follow-up chest computerized tomography, obtained after prolonged treatment for pneumonia. Histopathologic examination and HMB-45 staining demonstrated the mass to be an extrarenal angiomyolipoma. A comprehensive literature review of ERAMLs as well as evidence-based care algorithm is provided.

Angiomyolipomas (AMLs) are common primary renal parenchymal tumors which comprise about 1% of all renal masses. In contrast, extrarenal angiomyolipomas (ERAMLs) represent extremely rare tumors with less than 60 reported cases since they were first described by Friis and Hjortrup in 1982 [1]. ERAMLs typically present as incidentalomas identified on imaging for other conditions. Typically, these tumors have prominent vascular pedicles and may present symptomatically with abdominal pain and hemorrhagic shock [2].

This paper discusses the case of a 39-year-old female who presented asymptomatically with a 19.3 cm × 13.5 cm × 10.7 cm left extrarenal retroperitoneal mass detected on a follow-up chest computerized tomography (CT) after prolonged treatment for pneumonia. Given the location and appearance of the mass, differential diagnosis included a retroperitoneal liposarcoma, leiomyosarcoma, lipoma, angiomyolipoma, adrenal adenocarcinoma, renal cell carcinoma, or leiomyoma with fatty change.

## 2. Case Report

**Figure 1:**
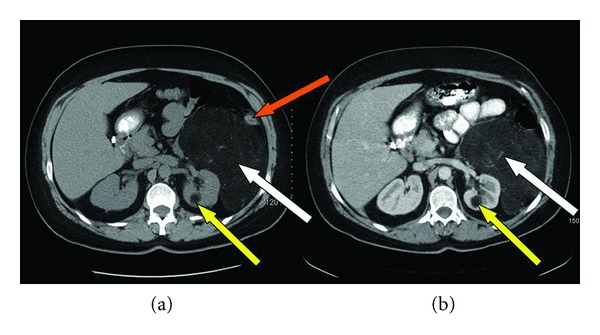
(a) oral contrast and (b) IV and oral contrast: abdominal computerized tomography demonstrating an encapsulated fatty vascular mass (white arrows) lateral to the left kidney measuring 19.3 cm × 13.5 cm × 10.7 cm with prominent vascular dependence on the left renal vein and artery as well as a 2 cm posterior mid-pole homogeneous fatty density (yellow arrow). Left colon is laterally displaced (orange arrow).

**Figure 2:**
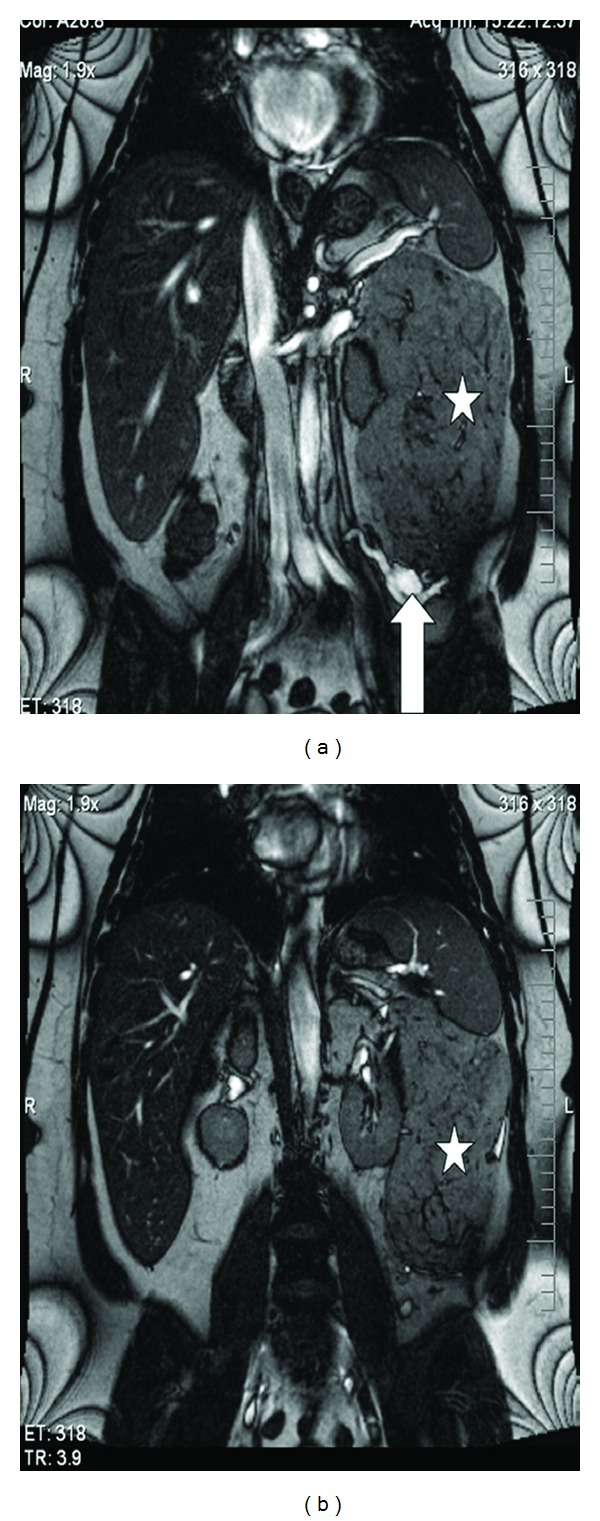
Abdominal magnetic resonance imaging demonstrating a large fatty encapsulated mass (white asterisk) measuring 19.3 cm × 13.5 cm × 10.7 cm with prominent vascularity (white arrows). The anatomic relationship between the mass and the left kidney can be well seen in Figure 2(b).

A 39-year-old female with a past medical and surgical history significant for hypertension, gastroesophageal reflux disease, and a cesarean section presented with dysfunctional uterine bleeding (DUB) in November, 2010. Transvaginal sonographic evaluation was performed and failed to reveal any gynecologic pathology. A dilatation and curettage were performed and she was started on oral contraceptives without resolution of her DUB. Two months later, she developed a protracted upper respiratory infection for which she was treated with a long course of antibiotics. Given the unexpected duration of her symptoms, a computerized tomography (CT) of the chest was obtained which was unremarkable; however, the lower CT images of the chest revealed a large retroperitoneal mass abutting the left kidney. A contrast-enhanced abdominal CT was subsequently obtained, which revealed an encapsulated mass measuring 19.3 cm × 13.5 cm × 10.7 cm with prominent vascularity arising from the left renal vasculature. In addition, there was a 2 cm inferior midpole homogeneous fatty renal lesion consistent with a renal angiomyolipoma (Figures 1(a) and 1(b)). A magnetic resonance image (MRI) of the abdomen was performed, demonstrating a fully encapsulated fatty tumor displacing the left colon laterally and measuring 19 cm × 14.4 cm × 13.8 cm. The mass tightly abutted the superior pole of the left kidney and a small 2 cm lesion inferiorly, most likely representing a renal angiomyolipoma, was also noted (Figures 2(a) and 2(b)).

Given the location of the lesion, the differential diagnosis included a retroperitoneal liposarcoma, leiomyosarcoma, lipoma, angiomyolipoma, adrenal adenocarcinoma, renal cell carcinoma, or leiomyoma with fatty change. The retroperitoneal mass was resected *en bloc* with the left kidney through a midline incision. A total abdominal hysterectomy (TAH) was simultaneously performed to treat her persistent DUB. The patient recovered uneventfully and was discharged home on postoperative day 6.

## 3. Pathology

**Figure 3:**
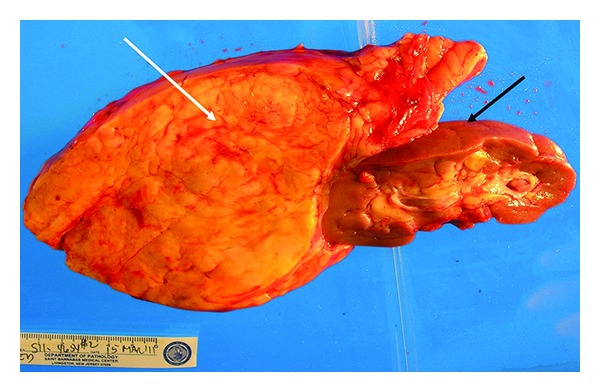
Gross image of the *en bloc* resected mass including the left kidney (black arrow), demonstrating a well-encapsulated fatty mass attached to the upper pole of the kidney (white arrow) with a smooth outer surface measuring 23 cm × 14 cm × 9 cm.

The *en bloc* gross specimen included the left kidney and weighed 1693 gm. The kidney measured 11.5 cm × 4.5 cm × 3 cm, and the mass located near the superior renal pole measured 23 cm × 14 cm × 9 cm (Figure 3). Serial sections of the mass and the kidney revealed it to be fully circumscribed and separate from the renal parenchyma. The mass was homogeneously yellow without stigmata of necrosis or hemorrhage. A second well-circumscribed, intra-renal mass, measuring 2 cm × 1.8 cm × 1 cm, was also identified within the inferior midportion of the renal cortex.

**Figure 4:**
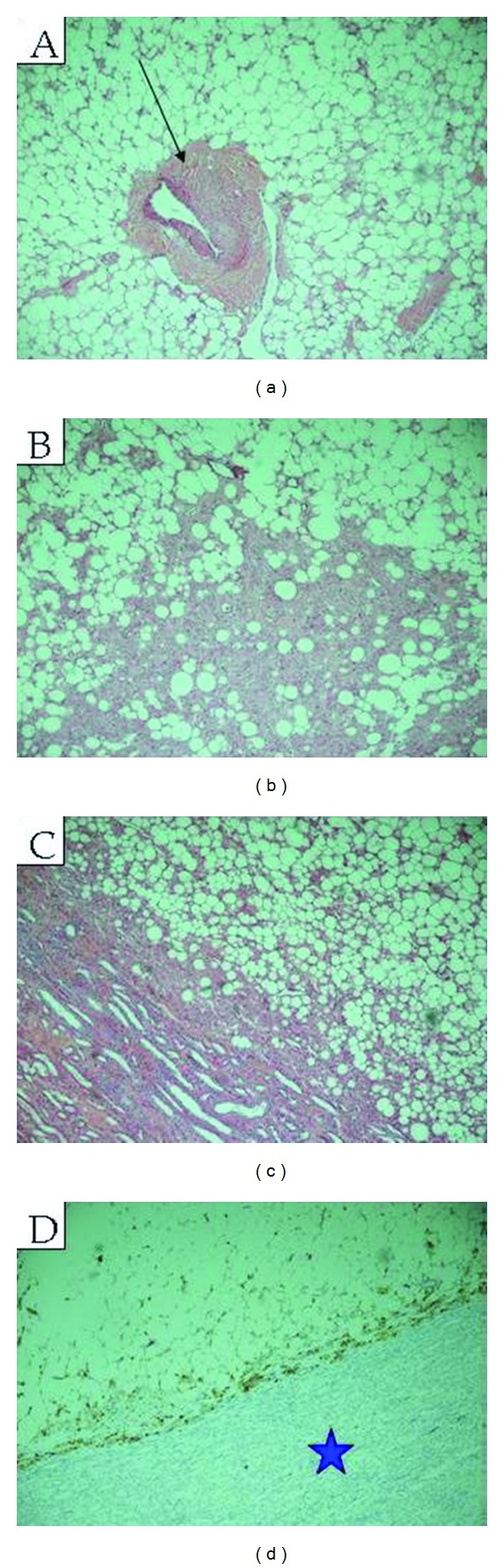
Extrarenal mass (Hematoxylin and Eosin). Photomicrograph of the mass demonstrate mature adipose tissue with a tortuous thick blood vessel (black arrow) ((a); ×20) and bundles of smooth muscles lacking elastic tissue lamina ((b); ×40), adipose tissue with small areas of smooth muscle with epithelioid features ((c); ×40). Focal staining with HMB45 antibody was positive (blue star) ((d); ×40), consistent with angiomyolipoma.

Both lesions had similar gross and microscopic features, with predominant adipose tissue and smaller areas of smooth muscle with epitheloid features and characteristic abnormal vessels. The larger lesion was distinct and separate from the renal parenchyma. HMB-45 staining performed on the larger lesion was positive, which is characteristic for an angiomyolipoma (Figure 4). Of note, the uterus and cervix had no microscopic abnormalities.

## 4. Discussion

Angiomyolipomas (AMLs) are rare complex mesenchymal neoplasms typically arising within the kidney and are composed of mature adipose tissue, smooth muscle cells, and thick-walled blood vessels [22]. Renal AMLs account for 1% of renal lesions, occurring more commonly in women [2] with an overall incidence in the general population of 0.07–0.3% [25]. Renal AMLs are sometimes referred to as hamartomas (a benign tumor-like growth composed of typical cells and tissues found in the area of the body where it occurs, but growing in a nonorganized fashion) or choristomas (a mass of normal tissue found in an ectopic location). Renal AMLs are generally felt to be more like a choristomas than hamartomas since kidneys do not normally contain smooth muscle or adipose cells [27, 28]. The presence of perivascular epithelioid cells (PEC) is often used to characterize angiomyolipomas since these cells show immunoreactivity for muscle markers (epithelial membrane antigen, keratin, vimentin, desmin, and actin) and HMB-45 [29]. Positive immunoreactivity for HMB-45, a monoclonal antibody raised against a melanoma-associated antigen, is characteristic of AMLs and can be used to differentiate AMLs from other similar appearing lesions such as liposarcomas, lipomas, leiomyosarcomas or, leiomyomas [20, 25].

Computerized tomography (CT) and computerized tomographic angiography (CTA) are the most commonly used imaging modalities to investigate AMLs. Wang et al. [28] analyzed the radiologic abdominal CTs characteristics of retroperitoneal extrarenal AMLs (ERAMLs) in an effort to distinguish them from liposarcomas. These authors noted that retroperitoneal ERAMLs typically display aneurysmal dilatation of the intratumor vessels, intratumoral linear vascularity, bridging veins, beak sign, hematomas, and discrete intrarenal/extrarenal fatty tumors, yet none of these are pathogneumonic. Magnetic resonance imaging (MRI), may also be used in conjunction with CT imaging and is particularly useful in delineating the anatomical relationship between ERAMLs, the kidney, and its vasculature, especially when dealing with perinephric and retroperitoneal AMLs. Brain CTs are recommended for patients with renal AMLs since 30–40% of these patients may also have features of tuberous sclerosis (TS) and similarly 80% of patients with TS will develop renal AML [30–32]. The brain CT of these patients typically demonstrates characteristic periventricular subependymal nodules with calcifications [2].

Surgery, and less often tumor embolization, is the primary treatment for ERAMLs. Surgical excision is indicated for symptomatic, complex appearing, radiologically enlarging, or large ERAMLs, which also have a higher tendency to bleed. In patients who present symptomatically with retroperitoneal hemorrhage, selective arterial embolization has been used effectively to control hemorrhagic lesions in hemodynamically unstable patients, resulting in tumor involution and subsequently allowing for elective resection or clinical observation [21, 23, 24]. Surgical resection is always advocated to differentiate suspected ERAMLs from other retroperitoneal lesions. Definitive diagnosis also dictates the length and type of appropriate followup since ERAMLs, unlike malignant retroperitoneal sarcomas or renal/adrenal carcinomas, may require long-term surveillance.

Distinct from renal AMLs, ERAMLs are extremely rare tumors with less than 60 reported cases worldwide in the literature. Friis and Hjortrup reported the first ERAML (1982) [1] involving a 22-year-old female presenting with abdominal pain and weight gain who was found on exploratory laparotomy to have an 11 kg retroperitoneal AML. Ditonno et al. [16] have reported the largest series of ERAMLs, involving 40 cases. In their report, the liver was the most common extrarenal location (*N* = 18), followed by the uterus (*N* = 7), retroperitoneum (*N* = 4), and head and vagina (*N* = 2 each) as well as one each involving the penis, nasal cavity, hard palate, abdominal wall, fallopian tube, spermatic cord, and colon. Other reported uncommon sites include the mediastinum, [33, 34] duodenum, appendix, stomach, and adrenal glands [9].

Retroperitoneal ERAMLs present a unique diagnostic challenge since they must be distinguished from other retroperitoneal masses including retroperitoneal sarcomas, atypical lipomas, adrenal adenocarcinomas, leiomyomas with fatty change, and renal cell carcinomas. Although the majority of ERAMLs are benign, 2 cases of metastatic and recurrent ERAMLs have been reported. Gupta et al. [26] described a case of a 29-year-old male with a history of tuberous sclerosis and a retroperitoneal AML which metastasized to the liver and mediastinum 19 years after initial diagnosis and resection. The second case involved an 80-year-old female who developed metastasis to liver and bone one year following surgical resection of a retroperitoneal AML. Although malignant transformation is difficult to predict, high mitotic activity within the primary tumor was a common factor in both metastatic cases. Additionally, certain ERAMLs variants, most notably the epitheloid variants, are thought to be the most aggressive, suggesting a higher likelihood of metastatic transformation and distant spread [26]. Rare cases of AML malignant transformation with lymph node involvement have been documented in the literature [18, 29–32]; however, all cases involved patients with renal AMLs and tuberous sclerosis.

To date, only 16 cases of retroperitoneal ERAMLs (including our case) have been reported, making the retroperitoneum the second most common extrarenal location of AMLs (Table 2). Among patients with retroperitoneal ERAMLs, the average age was 45 years (ranging from 22 to 80 yrs) with a male: female ratio of 1 to 5.3. Sixty-nine percent of patients with retroperitoneal ERAMLs presented symptomatically with nonspecific abdominal pain, 13% presented with incidentalomas, and another 13% with abdominal fullness. The most common imaging modality used to identify the ERAMLs was a CT scan (94% of cases). Retroperitoneal ERAMLs differed widely with respect to size, ranging from 6 cm^3^ to 7980 cm^3^ and weighing between <1 kg and 11 kgs. The majority of cases (69%) were managed surgically via *en bloc* radical nephrectomies and in 4 cases a renal-sparing resection was performed. One case was managed with embolization without resection.

56% of patients had follow-up evaluation ranging from 2 to 60 months after surgical resection. Outside the context of tuberous sclerosis, the only reported recurrence to date happened after a radical *en bloc* nephrectomy with distal metastasis to liver and bone 12 months postoperatively [26]. All other patients have remained disease-free and asymptomatic at last follow-up and no recurrence has been documented after a renal sparing nephrectomy or embolization. To date, the longest documented followup duration is 5 years, with that patient being asymptomatic and without a recurrence. As the only recurrence was documented 12 months after an *en bloc* radical nephrectomy, these lesions should be followed closely with CT imaging during the first year after resection, with continued yearly followup for 5 years or dictated by symptoms.

## 5. Conclusion

Extrarenal angiomyolipomas are rare and occur most commonly in the liver; however, the retroperitoneum is the second most common location. Lesions in the retroperitoneum present a unique diagnostic challenge since they can mimic other retroperitoneal benign and malignant tumors, which must be differentiated. CT scans and MRIs are the diagnostic imaging modalities of choice and are useful in delineating the anatomical relationship of these lesions to the kidney and its vasculature. Hemodynamically stable patients should undergo surgical resection, while unstable patients may benefit from emergent tumor embolization and a subsequently staged surgical resection. Once the pathology specimen is obtained, immunoreactivity to an HMB-45 stain is a useful tool to differentiate ERAMLs from other retroperitoneal tumors. ERAMLs should be analyzed from mitotic index and the presence of epitheloid variant as these characteristics may be associated with distal metastasis and disease recurrence. To date, only one case of disease recurrence and distal metastasis has been documented in a patient with tuberous sclerosis. This occurred in an 80-year-old female one year after a radical *en bloc* nephrectomy. Since the longest documented follow-up duration for an ERAML is 5 years, it is recommended that patients undergo serial CT imaging for the first year and be followed for 5 years based on symptoms. The ERAML reported in this paper measured 23 cm × 14 cm × 9 cm (2898 cm^3^), weighed 1.7 kgs (Table 1), and did not have increased mitotic index or epitheloid variance. Follow-up CT scans every 4 months revealed no recurrence and patient has remained disease-free 16 months postoperatively.

## Figures and Tables

**Table 1:** All reported cases of extrarenal angiomyolipomas (1982–2011).

Author	Location	*N*	Average age	Presenting symptoms
Demopoulos et al. [3]	Uterus	7	51	Pelvic pain, menometrorrhagia
Gutmann et al. [4]	Hard palate	1	39	Oral swelling
Bures and Barnes [5]	Head	2	25	Enlarging mass
Chen and Bauer [6]	Abdominal wall	1	42	Stress incontinence/abdominal pressure
Chaitin et al. [7]	Penis	1	53	Painless mass
Katz et al. [8]	Fallopian tube	1	40	Pelvic pain, menometrorrhagia
Miyahara et al. [9]	Liver	18	50	Epigastric tenderness
Dawlatly et al. [10]	Nasal cavity	1	52	Nasal obstruction/epistaxis
Peh and Sivanesaratnam [11] and Chen [12]	Vagina	2	46	Lower abdominal swelling
Castillenti and Bertin [13]	Spermatic cord	1	26	Testicular pain/scrotal swelling
Hikasa et al. [14]	Colon	1	67	melena
Current case, Minja et al.	Retroperitoneum	16	46	Abdominal pain, flank pain

Total		52	44.75 (mean)	

**Table 2:** Detailed information on all published retroperitoneal extrarenal angiomyolipomas (1982–2011).

Case	Author	Presenting symptoms	Age/sex	Size	Imaging	Location	Treatment	Followup/outcome
1	Friis and Hjortrup (1982) [1]	Pain, weight gain	22F	11 kg	IVU	PPS	RN	36 MO/asymptomatic
2	Randazzo et al. (1987) [15]	Pain, bleeding	64F	6 cm^3^	IVU, CT	Right PNS	RSR	2 MO/asymptomatic
3	Ditonno et al. (1992) [16]	Pain, bleeding	37M	5 cm	IVU, CT, A	Right PNS	RN	N/A^∗^
4	Peh et al. (1994) [17]	Weight loss/abdominal mass	32F	3.7 kg (7980 cm^3^)	US, CT	Left PNS	RN	8 MO/asymptomatic
5	Angulo et al. (1994) [18]	Abdominal pain, flank pain	53F	336 cm^3^	US, CT, A	Left PNS	RN	N/A^∗^
6	Gupta and Guleria (2011) [19]	Abdominal pain	42M	220 cm^3^	US, CT	Right AS	RSR	N/A^∗^
7	Liwnicz et al. (1994) [20]	Abdominal pain	39F	1.1 kg (216 cm^3^)	CT	Right PNS	RN	18 MO/asymptomatic
8	Law et al. (1994) [21]	Incidental finding	59F	22.5 cm^3^	CT, MRI	Left PNS	RN	N/A^∗^
9	Law et al. (1994) [21]	Pain	56F	11 cm	IVU, CT, US, FNA	Left PNS	RN	8 MO/asymptomatic
10	Mogi et al. (1998) [22]	Abdominal pain + fullness	41F	648 cm^3^	CT, MRI	Right PNS/PHS	RSR	N/A^∗^
11	Murphy et al. (2000) [23]	Abdominal pain, bleeding	51F	ND	CT, A	Left PNS	AE	12 MO/asymptomatic
12	Tsutsumi et al. (2001) [2]	Fatigue, abdominal pain	60F	3.5 kg (4840 cm^3^)	CT, A	Right PNS	RN	60 MO/asymptomatic
13	Tseng et al. (2004) [24]	Abdominal fullness	35F	2.8 kg (3726 cm^3^)	US, CT, A	Right PNS	RSR	N/A^∗^
14	Obara et al. (2005) [25]	Macroscopic hematuria	31M	ND	CT, A	Right PNS	RN	N/A^∗^
15	Gupta et al. (2007) [26]	Abdominal pain	80F	16 cm	CT, MRI	Left PNS	RN	1 year/distal metastases
16	Our Case (2011)	Asymptomatic	39F	1.7 kg (2898 cm^3^)	US, CT	Left PNS	RN	16 MO/asymptomatic

ND: not documented; A: angiography; CT: computerized tomography; MRI: magnetic resonance imaging; US: ultrasound; IVU: intravenous urography; FNA: fine needle aspiration; PNS: perinephric space; PHS: perihepatic space; PPS: peripancreatic space; AS: adrenal space; RN: radical nephrectomy; RSR: renal sparing resection; AE: angio embolization; MO: months; N/A^∗^: follow-up information not available.
